# Mathematical Modeling and Experimental Investigation of the Dynamic Response for an Annular Circular Plate Made of Glass/Polyester Composite Under Different Boundary Conditions

**DOI:** 10.1155/2024/4022144

**Published:** 2024-09-16

**Authors:** Riyadh Makki Hashim, Samaher Mohammed Sarhan, Fathi Al-Shamma, M. N. Mohammed, Salah Al-Zubaidi, Oday I. Abdullah

**Affiliations:** ^1^ Department of Automated Manufacturing Engineering Al-Khwarizmi College of Engineering University of Baghdad, Baghdad 10071, Iraq; ^2^ Department of Mechatronics Engineering Al-Khwarizmi College of Engineering University of Baghdad, Baghdad 10071, Iraq; ^3^ Department of Mechanical Engineering College of Engineering University of Baghdad, Baghdad, Iraq; ^4^ Mechanical Engineering Department College of Engineering Gulf University, Sanad 26489, Bahrain; ^5^ Department of Energy Engineering College of Engineering University of Baghdad, Baghdad 10071, Iraq; ^6^ College of Engineering Gulf University, Sanad 26489, Bahrain; ^7^ Department of Mechanics Al-Farabi Kazakh National University, Almaty 050040, Kazakhstan

**Keywords:** annular circular plate, composite material, dynamic analysis, vibration

## Abstract

Fiber-reinforced elastic laminated composites are extensively used in several domains owing to their high specific stiffness and strength and low specific density. Several studies were performed to ascertain the factors that affect the composite plates' dynamic properties. This study aims to derive a mathematical model for the dynamic response of the processed composite material in the form of an annular circular shape made of polyester/E-glass composite. The mathematical model was developed based on modified classical annular circular plate theory under dynamic loading, and all its formulas were solved using MATLAB 2023. The mathematical model was also verified with real experimental work involving the vibration test of the fabricated composite plate. The composite plate was processed by reinforcing the polyester matrix with E-glass fibers with a 50% volume fraction each by using the handy lay-up method. After fabrication, the composite plate was tested with a universal vibration tester, where the plate was impacted and released to free vibration, and the deflection was measured experimentally to compare it with the theoretical value calculated from the derived model. The plate was tested under two boundary conditions, namely, simply and built-in supported. The findings show good agreement between theoretical and experimental plate deflections at different angles, particularly at built-in supported boundary conditions. Also, a higher natural frequency was recorded at this condition compared to others, and this may be ascribed to the higher shear stresses involved due to large moments at the ends along with supporting. Meanwhile, the real experimental spectrum of the built-in condition was higher than others, as the sig view curve revealed.

## 1. Introduction

Fiber-strengthened elastic laminated composites are increasingly being adopted by aerospace and other industries owing to the material's superior specific strength, stiffness, and low specific density [1]. Composites are shaped as annular circular plates and are used for aerospace applications, submarines, pressure containers, and roof domes [2]. The annular laminated composite is utilized in many complex industrial structures. Hence, the unconstrained vibration of composite plates having a circular hole cap used in high-pressure pump tankers is a critical issue from the fundamental frequency standpoint. Therefore, studying the vibration behavior of annular composite plates is meaningful and fruitful in terms of noise reduction and impact behavior. Numerous investigations were conducted to develop new approaches or improve the available ones to solve vibration problems of mechanical structures. They involve classical plate theory (CPT), zigzag theory, 3D elastic theory, layerwise theory, first-order shear deformation theory (FSDT), and higher order shear deformation theory (HSDT), and others.

In the manufacturing of the structure of air space frames such as the tip of the front of the fuselage airplane, the rockets or other space ships are needed in the design considering the light of the weight with respect to the high strength of the structure and to withstand the impulse dynamic loading that effect on the front face which constructed from different size of annular plates. Several studies have been conducted in this field to develop analytical and numerical solutions for studying the dynamic response of composite materials in different geometries and under different boundary conditions.

For instance, the force vibration of the composite laminated annular plate under the Winkler foundation was solved by Afsharmanesh, Ghaheri, and Taheri-Behrooz [3] based on CPT and Ritz methods. Rayleigh–Ritz method was adopted by Amabili, Pierandrei, and Frosali [4] to solve free vibration of the riveted annular composite under elastic boundary conditions. Mercan, Baltacıoglu, and Civalek [5] applied FSDT and discrete singular convolution (DSC) methods to find the significant vibration variables of annular and conical plates made of isotropic FG composite laminated materials. Also, Amabili et al. [6–9] conducted multiple studies on the forced nonlinear vibration of the composite plate by using third-order shear deformation theory. Khare and Mittal [10] evaluated the free vibration characteristics of laminated composite annular plates under stressful hydrothermal conditions. The Green–Lagrange nonlinear kinematics approach was employed to devise the mathematical framework for the laminated annular plate based on the higher order shear deformation concept.

Jin et al. [11] contrasted the two- and three-dimensional (2D vs. 3D) finite elements (FEs), refined and conventional 2D generalized differential quadrature (GDQ) techniques along with a precise three-dimensional proposal to evaluate free vibrations concerning single- and multilayered isotropic composite annular composite plates. Amabili, Pierandrei, and Frosali [4] evaluated the free vibration challenges concerning laminated composite stiffened circular plates having cutouts based on several boundary criteria based on the FE technique. Arshid and Khorshidvand [12] performed a precise free vibration assessment concerning doubly curved laminated composite annular circular plate based on piezoelectric techniques by integrating the dynamic stiffness method (DSM) and the HSDT.

Mercan, Baltacıoglu, and Civalek [5] provided mathematical outcomes concerning laminated composite plate/shell structures based on the FSDT approach to study shear disfigurement using the DSC technique. Viswanathan et al. [13] evaluated free vibrations related to composite-based spherical shell caps without and with a cutout. The authors used the FE technique associated with the higher order shear deformation concept. Powmya and Narasimhan [14] evaluated the free vibration properties pertaining to a rotating laminated shell based on Chebyshev collocation. The outcomes were reported primarily for the impact loading scenario.

Wilson [15] used the FE technique to report the free vibration properties pertaining to a composite-based doubly curved laminate subjected to impact loading. The analysis relies on the first-order shear deformation concept pertaining to the laminated doubly curved circular sheet. Katariya, Panda, and Mehar [16] evaluated a practical technique for conceptual modeling and practical validation of modal outcomes pertaining to skewed laminated sandwich builds having a soft epoxy-based core; they assessed the free vibration properties of an orthotropic annular spherical sheet having clamped boundary criteria along all edges.

Bounouara et al. [17] studied the linear, nonlinear, and uniform thermal stresses with constant mechanical loading of anisotropic composite materials on the visco-Pasternak along the plate thickness. The study involved investigating the influence of geometric ratio, the ratio of the coefficient of thermal expansion, the coefficient of damping, and foundation variables on the thermomechanical bending behavior of thick plate composite materials. Belbachir et al. [18] employed a refined quasi-3D trigonometric shear deformation method to study the stability and dynamic behaviors of laminated composite plates. Four unknowns were included in the displacement field, besides considering the thickness effect and involving the mathematical model with the Winkler–Pasternak elastic base. Good accuracy was demonstrated when the model results were compared with other literature.

Based on the reviewed works above, it becomes more complicated when utilizing different layers of composite material with different volumetric ratios especially in annular circular plates with different boundary conditions so that the main performance that gives a special performance for the design of the structure especially in the kind of the transient vibration causes good illustration of the factors that affect the design of the structure. Therefore, the current study aims to present mathematical modeling of free vibration for the orthotropic annual circular plate under impact loading and different boundary conditions. The mathematical model was derived based on the modified classical annular circular plate theory and validated with a real experimental investigation for the processed glass/polyester composite material. The processed plate was subjected to clamped, partially supported, and simply supported boundary conditions.

## 2. Deriving the Mathematical Model

The typical circular plate equation is stated as a function of its coordinates (*r* and *θ*) [19]

(1) $$\begin{array}{c} \left(\frac{\partial^{2}}{{\partial r}^{2}}+\frac{2}{r}\frac{\partial }{\partial r}\right)*M_{r}+\left(-\frac{1}{r}\frac{\partial }{\partial r}+\frac{1}{r^{2}}\frac{\partial^{2}}{{\partial \theta }^{2}}\right)*M_{\theta }+\left(-\frac{2}{r}\frac{\partial^{2}}{\partial r\partial \theta }-\frac{2}{r^{2}}\frac{\partial }{\partial \theta }\right)*M_{r\theta }+q=0. \end{array}$$

The bending and twist moments for the orthotropic plate are specified as follows:

(2) $$\begin{array}{c} M_{r}=-\left[D_{r}*\frac{\partial^{2}w}{{\partial r}^{2}}+D_{1}*\left(\frac{1}{r}\frac{\partial w}{\partial r}+\frac{1}{r^{2}}\frac{\partial^{2}w}{{\partial \theta }^{2}}\right)\right], \end{array}$$

(3) $$\begin{array}{c} M_{r\theta }=2*\left[\frac{1}{r}\frac{\partial^{2}w}{\partial r\partial \theta }-\frac{1}{r^{2}}\frac{\partial w}{\partial \theta }\right]*D_{r\theta }, \end{array}$$

and

(4) $$\begin{array}{c} D_{r}=\frac{E_{r}*t^{3}}{12*\left(\nu_{r\theta }*\nu_{\theta r}\right)},\\ D_{\theta }=\frac{E_{\theta }*t^{3}}{12*\left(\nu_{r\theta }*\nu_{\theta r}\right)},\\ D_{1}=\frac{\nu_{r\theta }*E_{\theta }*t^{3}}{12*\left(\nu_{r\theta }*\nu_{\theta r}\right)}=\frac{\nu_{\theta r}*E_{r}*t^{3}}{12*\left(\nu_{r\theta }*\nu_{\theta r}\right)},\\ D_{r\theta }=\frac{\sqrt{D_{r}*D_{\theta }}*\left(1-\sqrt{\nu_{r\theta }*\nu_{\theta r}}\right)}{2}. \end{array}$$

The first term in equation (1) becomes

(5) $$\begin{array}{c} \left(\frac{\partial^{2}}{{\partial r}^{2}}+\frac{2}{r}\frac{\partial }{\partial r}\right)*M_{r}=-\;\left(D_{r}*\frac{\partial^{4}w}{{\partial r}^{4}}+\frac{D_{1}}{r}\frac{\partial^{3}w}{{\partial r}^{3}}+\frac{D_{1}}{r^{2}}\frac{\partial^{4}w}{{\partial r}^{2}{\partial \theta }^{2}}-\frac{2*D_{1}}{r^{3}}\frac{\partial^{3}w}{{\partial r\partial \theta }^{2}}+\frac{2*D_{1}}{r^{4}}\frac{\partial^{2}w}{{\partial \theta }^{2}}+\frac{2*D_{r}}{r}\frac{\partial^{3}w}{{\partial r}^{3}}\right). \end{array}$$

The second term in equation (1) becomes

(6) $$\begin{array}{c} \left(-\frac{1}{r}\frac{\partial }{\partial r}+\frac{1}{r^{2}}\frac{\partial^{2}}{{\partial \theta }^{2}}\right)*M_{\theta }=-\;\left(-\frac{D_{\theta }}{r^{2}}\frac{\partial^{2}w}{{\partial r}^{2}}+\frac{D_{\theta }}{r^{3}}\frac{\partial w}{\partial r}+\frac{2*D_{\theta }}{r^{4}}\frac{\partial^{2}w}{{\partial \theta }^{2}}-\frac{D_{1}}{r}\frac{\partial^{3}w}{{\partial r}^{3}}+\frac{D_{\theta }}{r^{4}}\frac{\partial^{4}w}{{\partial \theta }^{4}}+\frac{D_{1}}{r^{2}}\frac{\partial^{4}w}{{\partial r}^{2}{\partial \theta }^{2}}\right). \end{array}$$

Further, the third term in equation (1) is stated as follows:

(7) $$\begin{array}{c} \left(-\frac{2}{r}\frac{\partial^{2}}{\partial r\partial \theta }-\frac{2}{r^{2}}\frac{\partial }{\partial \theta }\right)*M_{r\theta }=2*D_{r\theta }\left(-\frac{2}{r^{2}}\frac{\partial^{4}w}{{\partial r}^{2}{\partial \theta }^{2}}+\frac{2}{r^{3}}\frac{\partial^{3}w}{\partial r{\partial \theta }^{2}}-\frac{2}{r^{4}}\frac{\partial^{2}w}{{\partial \theta }^{2}}\right). \end{array}$$

The value can be substituted concerning the three terms in equation (1), leading to the following expression:

(8) $$\begin{array}{c} D_{r}*\frac{\partial^{4}w}{{\partial r}^{4}}+\frac{2*D_{r}}{r}\frac{\partial^{3}w}{{\partial r}^{3}}-\frac{D_{\theta }}{r^{2}}\frac{\partial^{2}w}{{\partial r}^{2}}+\frac{D_{\theta }}{r^{3}}\frac{\partial w}{\partial r}+\frac{2*\left(D_{1}+2*D_{r\theta }\right)}{r^{2}}\frac{\partial^{4}w}{{\partial r}^{2}{\partial \theta }^{2}}-\frac{2*\left(D_{1}+2*D_{r\theta }\right)}{r^{3}}\frac{\partial^{3}w}{\partial r{\partial \theta }^{2}}+\frac{2*\left(D_{1}+D_{\theta }+2*D_{r\theta }\right)}{r^{4}}\frac{\partial^{2}w}{{\partial \theta }^{2}}+\frac{D_{\theta }}{r^{4}}\frac{\partial^{4}w}{{\partial \theta }^{4}}=q. \end{array}$$

The homogeneous solution of equation (1) is specified as follows:

(9) $$\begin{array}{c} {w\left(r,\theta \right)}_{H}=A*\sin\;\sin\left(r*\theta \right)+B*\cos\left(r*\theta \right), \end{array}$$

where A and B are constants.

The homogeneous solutions (1), (2), (8), and (9) can be determined for *r* and *θ*:

(10) $$\begin{array}{c} \frac{\partial w}{\partial r}=A*\theta *\cos\left(r*\theta \right)-B*\theta *\sin\left(r*\theta \right),\\ \frac{\partial^{2}w}{{\partial r}^{2}}=-A*\theta^{2}*\sin\left(r*\theta \right)-B*\theta^{2}*\cos\left(r*\theta \right),\\ \frac{\partial^{3}w}{{\partial r}^{3}}=-A*\theta^{3}*\cos\left(r*\theta \right)+B*\theta^{3}*\sin\left(r*\theta \right),\\ \frac{\partial^{4}w}{{\partial r}^{4}}=A*\theta^{4}*\sin\left(r*\theta \right)+B*\theta^{4}*\cos\left(r*\theta \right),\\ \frac{\partial^{4}w}{{\partial r}^{2}{\partial \theta }^{2}}=-A*\left(-\theta^{2}*r^{2}*\sin\;\sin\left(r*\theta \right)+4*\theta *r*\cos\left(r*\theta \right)+2*\sin\left(r*\theta \right)\right)-B*\left(-\theta^{2}*r^{2}*\cos\;\cos\left(r*\theta \right)-4*\theta *r*\sin\left(r*\theta \right)+2*\cos\left(r*\theta \right)\right),\\ \frac{\partial^{3}w}{\partial r{\partial \theta }^{2}}=A*\left(-\theta *r^{2}*\cos\left(r*\theta \right)-2*r*\sin\left(r*\theta \right)\right)-B*\left(-\theta *r^{2}*\sin\left(r*\theta \right)+2*r*\cos\left(r*\theta \right)\right),\\ \frac{\partial^{2}w}{{\partial \theta }^{2}}=-A*r^{2}*\sin\left(r*\theta \right)-B*r^{2}*\cos\left(r*\theta \right),\\ \frac{\partial^{4}w}{{\partial \theta }^{4}}=A*r^{4}*\sin\left(r*\theta \right)+B*r^{4}*\cos\left(r*\theta \right). \end{array}$$

These expressions are substituted in equation (1), and the simplified expression is as follows:

(11) $$\begin{array}{c} w{\left(r,\theta \right)}_{H}=\frac{\left(2*D_{r}*\theta^{3}*r^{2}+6*D_{1}*r^{2}*\theta +12*D_{r\theta }*r^{2}*\theta -D_{\theta }*\theta \right)}{\left(D_{r}*\theta^{4}*r^{3}+D_{\theta }*\theta^{2}*r+2*D_{1}*r^{3}*\theta^{2}+4*D_{r\theta }*r^{3}*\theta^{2}-2*D_{1}*r-2*D_{\theta }*r-4*D_{r\theta }*r+r^{3}\right)}*A*\cos\left(r*\theta \right)-\frac{\left(2*D_{r}*\theta^{3}*r^{2}+6*D_{1}*r^{2}*\theta +12*D_{r\theta }*r^{2}*\theta -D_{\theta }*\theta \right)}{\left(D_{r}*\theta^{4}*r^{3}+D_{\theta }*\theta^{2}*r+2*D_{1}*r^{3}*\theta^{2}+4*D_{r\theta }*r^{3}*\theta^{2}-2*D_{1}*r-2*D_{\theta }*r-4*D_{r\theta }*r+r^{3}\right)}*B*\sin\left(r*\theta \right). \end{array}$$

And the specific solution is as follows:

(12) $$\begin{array}{c} w{\left(r,\;\theta \right)}_{P}=C*r*\theta . \end{array}$$

Substitute equation (12) in plate equation (1) and determine constant C:

(13) $$\begin{array}{c} C=\frac{q*r^{3}}{\theta *D_{\theta }},\\ w{\left(r,\theta \right)}_{P}=\frac{q*r^{4}}{D_{\theta }}.\; \end{array}$$

The complementary solution concerning deflection is specified as follows:

(14) $$\begin{array}{c} w\left(r,\theta \right)=w{\left(r,\theta \right)}_{H}+w{\left(r,\theta \right)}_{P},\\ w\left(r,\theta \right)=\frac{\left(2*D_{r}*\theta^{3}*r^{2}+6*D_{1}*r^{2}*\theta +12*D_{r\theta }*r^{2}*\theta -D_{\theta }*\theta \right)}{\left(D_{r}*\theta^{4}*r^{3}+D_{\theta }*\theta^{2}*r+2*D_{1}*r^{3}*\theta^{2}+4*D_{r\theta }*r^{3}*\theta^{2}-2*D_{1}*r-2*D_{\theta }*r-4*D_{r\theta }*r+r^{3}\right)}*A*\cos\left(r*\theta \right)-\frac{\left(2*D_{r}*\theta^{3}*r^{2}+6*D_{1}*r^{2}*\theta +12*D_{r\theta }*r^{2}*\theta -D_{\theta }*\theta \right)}{\left(D_{r}*\theta^{4}*r^{3}+D_{\theta }*\theta^{2}*r+2*D_{1}*r^{3}*\theta^{2}+4*D_{r\theta }*r^{3}*\theta^{2}-2*D_{1}*r-2*D_{\theta }*r-4*D_{r\theta }*r+r^{3}\right)}*B*\sin\left(r*\theta \right)+\frac{q*r^{4}}{D_{\theta }}. \end{array}$$

It can be used for the boundary criteria in equation (1) to determine constants A and B

At *r*=*r*_1_=2.5 cm,  *θ*=*θ*_1_,  *w*(*r*,  *θ*)=0. At *r*=*r*_1_=2.5 cm,  *θ*=*θ*_2_,  *w*(*r*,  *θ*)=0. *θ*_1_ and *θ*_2_ differ for every flank.

Then,

(15) $$\begin{array}{c} A=\frac{C_{1}*C_{3}*\sec\;\sec\left(r_{1}*\theta_{2}\right)*\cos\;\cos\left(r_{1}*\theta_{1}\right)*\tan\;\tan\left(r_{1}*\theta_{2}\right)-C_{2}*C_{3}*\tan\left(r_{1}*\theta_{2}\right)}{C_{1}*C_{2}*\left(\tan\;\tan\left(r_{1}*\theta_{2}\right)*\cos\;\cos\left(r_{1}*\theta_{1}\right)-\;\sin\;\sin\left(r_{1}*\theta_{1}\right)\right)}-\frac{C_{3}*\sec\;\sec\left(r_{1}*\theta_{2}\right)\;}{C_{2}},\\ B=\frac{C_{1}*C_{3}*\sec\;\sec\left(r_{1}*\theta_{2}\right)\left(r_{1}*\theta_{1}\right)-C_{2}*C_{3}}{C_{1}*C_{2}*\left(\tan\;\tan\left(r_{1}*\theta_{2}\right)*\cos\;\cos\left(r_{1}*\theta_{1}\right)-\;\sin\;\sin\left(r_{1}*\theta_{1}\right)\right)}, \end{array}$$

where

(16) $$\begin{array}{c} C_{1}=\frac{2*D_{r}*\theta_{1}^{3}*r_{1}^{2}+6*D_{1}*r_{1}^{2}*\theta_{1}+12*D_{r\theta }*r_{1}^{2}*\theta_{1}-D_{\theta }*\theta_{1}}{D_{r}*\theta_{1}^{4}*r_{1}^{3}+D_{\theta }*\theta_{1}^{2}*r_{1}+2*D_{1}*r_{1}^{3}*\theta_{1}^{2}+4*D_{r\theta }*r_{1}^{3}*\theta_{1}^{2}-2*D_{1}*r_{1}-2*D_{\theta }*r_{1}-4*D_{r\theta }*r_{1}+r_{1}^{3}},\\ C_{2}=\frac{2*D_{r}*\theta_{2}^{3}*r_{1}^{2}+6*D_{1}*r_{1}^{2}*\theta_{2}+12*D_{r\theta }*r_{1}^{2}*\theta_{2}-D_{\theta }*\theta_{2}}{D_{r}*\theta_{2}^{4}*r_{1}^{3}+D_{\theta }*\theta_{2}^{2}*r_{1}+2*D_{1}*r_{1}^{3}*\theta_{2}^{2}+4*D_{r\theta }*r_{1}^{3}*\theta_{2}^{2}-2*D_{1}*r_{1}-2*D_{\theta }*r_{1}-4*D_{r\theta }*r_{1}+r_{1}^{3}},\\ C_{3}=\frac{q*r_{1}^{4}}{D_{\theta }},\\ ∴w\left(r,\theta \right)=\frac{\left(2*D_{r}*\theta^{3}*r^{2}+6*D_{1}*r^{2}*\theta +12*D_{r\theta }*r^{2}*\theta -D_{\theta }*\theta \right)}{\left(D_{r}*\theta^{4}*r^{3}+D_{\theta }*\theta^{2}*r+2*D_{1}*r^{3}*\theta^{2}+4*D_{r\theta }*r^{3}*\theta^{2}-2*D_{1}*r-2*D_{\theta }*r-4*D_{r\theta }*r+r^{3}\right)}*\left[\frac{C_{1}*C_{3}*\sec\;\sec\left(r_{1}*\theta_{2}\right)*\cos\;\cos\left(r_{1}*\theta_{1}\right)*\tan\;\tan\left(r_{1}*\theta_{2}\right)\;-C_{2}*C_{3}*\tan\;\tan\left(r_{1}*\theta_{2}\right)\;}{C_{1}*C_{2}*\left(\tan\;\tan\left(r_{1}*\theta_{2}\right)*\cos\;\cos\left(r_{1}*\theta_{1}\right)\;-\;\sin\;\sin\;\left(r_{1}*\theta_{1}\right)\;\right)}-\frac{C_{3}*\sec\;\sec\left(r_{1}*\theta_{2}\right)\;}{C_{2}}\right]*\cos\;\cos\left(r*\theta \right)\;-\frac{\left(2*D_{r}*\theta^{3}*r^{2}+6*D_{1}*r^{2}*\theta +12*D_{r\theta }*r^{2}*\theta -D_{\theta }*\theta \right)}{\left(D_{r}*\theta^{4}*r^{3}+D_{\theta }*\theta^{2}*r+2*D_{1}*r^{3}*\theta^{2}+4*D_{r\theta }*r^{3}*\theta^{2}-2*D_{1}*r-2*D_{\theta }*r-4*D_{r\theta }*r+r^{3}\right)}*\left[\frac{C_{1}*C_{3}*\sec\;\sec\left(r_{1}*\theta_{2}\right)\;\left(r_{1}*\theta_{1}\right)\;-C_{2}*C_{3}}{C_{1}*C_{2}*\left(\tan\;\tan\left(r_{1}*\theta_{2}\right)*\cos\;\cos\;\left(r_{1}*\theta_{1}\right)\;-\;\sin\;\sin\left(r_{1}*\theta_{1}\right)\;\right)}\right]*\sin\;\sin\left(r*\theta \right)+\frac{q*r^{4}}{D_{\theta }}, \end{array}$$

It may be said that the two contact load coordinates are equivalent to the simply supported beam having a point load (*q*) per unit length based on the dynamic impact concept. The impact velocity change rate is defined as follows:

(17) $$\begin{array}{c} m_{1}\frac{dv_{1}}{dt}=-q. \end{array}$$

If we use an identical distance, the target and impactor approach due to local compression at the contact point, leading to the approach velocity being

(18) $$\begin{array}{c} \mu^{\cdot }=v_{1}+v_{2}. \end{array}$$

If the impact is for an extended duration, the Hertzian law may be used to derive

(19) $$\begin{array}{c} q=n_{1}\mu^{3/2}, \end{array}$$

where

(20) $$\begin{array}{c} n_{1}=\frac{4\surd R_{1}}{3\pi \left(k_{1}+k_{2}\right)}. \end{array}$$

*k*_1_ and *K*_2_ are constants based on impactor and target characteristics. The final equation after differentiating equation (18) and merging with equation (1), followed by substitution of equation (19), produces

(21) $$\begin{array}{c} \mu^{\cdot \cdot }=-\frac{h_{1}}{m_{1}}\mu^{3/2}. \end{array}$$

The two sides of equation (21) are multiplied by *μ*^∙^, and the expression is integrated:

(22) $$\begin{array}{c} {\mu^{\cdot }}^{2}-V^{2}=-\frac{4}{5}\frac{n_{1}\mu^{5/2}}{m_{1}}, \end{array}$$

where *V* denotes the approach velocity concerning the two bodies at *t* = 0 when impact starts; hence, maximum deformation (*μ*_1_) occurs at *μ*^∙^=0 and is specified as follows:

(23) $$\begin{array}{c} \mu_{1}={\left[\frac{5m_{1}V^{2}}{4n_{1}}\right]}^{2/5}. \end{array}$$

Substituting of equation (23) into equation (19) gives

(24) $$\begin{array}{c} q={n_{1}}^{2/5}{\left[\frac{5m_{1}V^{2}}{4}\right]}^{3/5}. \end{array}$$

The potential energy expression uses this *q* value, leading to energy being a function of initial impact velocity, mass, and plate characteristics and dimensions. Hence, the fundamental frequency is computed using potential energy, and the values provide varying shape factors concerning the natural frequency.

All the above equations have been solved by using MATLAB 2023 by applying the following steps:

• For the orthotropic plate, find the value of *M*_*r*_, *M*_*θ*_, and *M*_*rθ*_ using equation (2) as a function of *w*.
• Find the plate rigidity (*D*_*r*_, *D*_*θ*_, and *D*_*rθ*_) using equation (2).
• Calculate the first, second, and third terms of equation (1) by substituting the values of *M*_*r*_, *M*_*θ*_, and *M*_*rθ*_, respectively.
• Take the values of these terms and substitute them in the moment equation (1) to derive the general equation of motion 3 as a function of *w*.
• Determine the homogeneous deflection Wh as a function of *r* and *θ* by taking the assumption of sin (*r*, *θ*) and cos (*r*, *θ*).
• Substitute this assumption of Wh in equation (1) to find its value as a function of (*r*, *θ*).
• Find the particular solution of Wp as a function of (*r*, *θ*).
• Apply the boundary conditions to calculate the constants: A, B, C1, C2, and C3, respectively.
• Derive the value of the load *q* as impact loading taking into account the dynamic impact loading equations (17)–(23).
• The final value of *q* is a function of time from equation (24) which depends on the velocity and type of contact between the two bodies.
• Derive the final characteristic equation by substituting the value of *q* to find the natural frequency.

Table 1 provides the definition and unit for all the variables and symbols used in deriving the mathematical model.

## 3. Experimental Analysis

In this study, a composite material has been fabricated from a polyester matrix-reinforced woven fiberglass and shaped in the form of an annual circular plate. The composite material was produced using a hand lay-up process employing male and female aluminum molds. The internal and external diameters of the half-male mold are 20 and 40 cm with 20 mm thickness.

**Figure 1:**
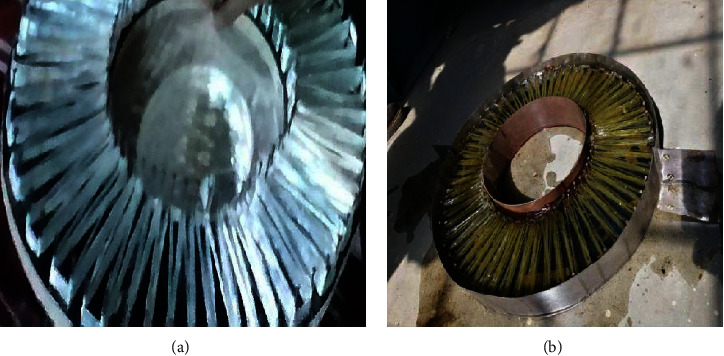
The fabricated circular composite plate: (a) male mold with distributed E-glass fibers before layering the resin and (b) the final part after resin layering.

Preparation of the orthotropic plate required the mold surface to be cleaned using acetone, followed by wax coating. The E-glass fibers were positioned and distributed radially with a 2-cm circular pitch and a 2.5-mm vertical pitch, as depicted in Figure 1. Later, the resin is layered evenly using a brush, followed by the initial mat layer laid using a steel roller over the fabric. The previous step improves impregnation and wetting; subsequently, the second mat layer is deposited. This process is used to lay all fabric layers, and the female mold is used to press the layers. Processing of the composite was performed at room temperature for about 8 hr, followed by removal from the mold to get a useable composite plate, as depicted in Figure 1.

### 3.1. Mechanical Properties

The standard mechanical characteristics of constituents of the test samples (E-glass orthotropic fibers and polyester matrix) are provided in Table 2. The composite shell has 1737.5 kg/m^3^ density, 50% volume fraction, 22-GPa elasticity modulus, and 0.27 Poisson ratio.

### 3.2. Vibration Test

**Figure 2:**
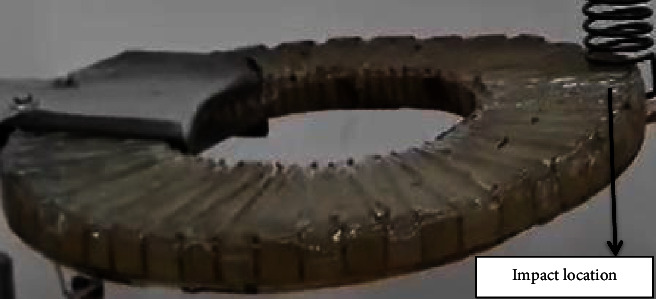
The three testing locations of the boundary conditions.

**Figure 3:**
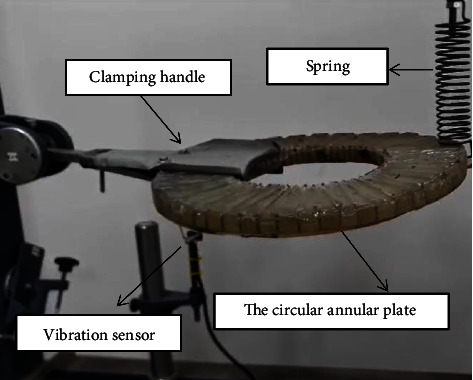
Vibration test setup for the orthotropic composite annular circular plate.

**Figure 4:**
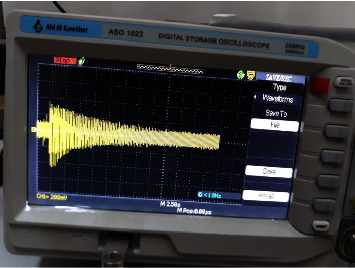
Test the wave that comes from the free vibration of the annular circular plate.

The fabricated orthotropic composite circular plate was subjected to a vibration test using a universal vibration tester. The circular plate was tested under two boundary conditions, namely, built and simply supported. The position of the impact location for both boundary conditions is illustrated in Figure 2. At each boundary condition, the location is impacted down and released to vibrate freely. A Piezo vibration sensor (type MEAS) was attached under the clamping as shown in Figure 3. The sensor, according to the manufacturer, can generate a voltage of up to 90 volts when it experiences a vibration or impact loading. The sensor is connected to an oscilloscope (type DS1102E). The oscilloscope generates a wave-like signal as shown in Figure 4 when the plate is allowed to vibrate freely after impacting and releasing.

## 4. Results and Discussions

This study aims to derive a mathematical model based on modified classical annular circular plate theory under dynamic loading and validated with real experimental work to ascertain the parameters that impact the natural frequency corresponding to annular circular composite plates. Hence, the dynamic characteristics should evaluate the high deflection value pertaining to the support criteria (boundary conditions). The change in the composite's natural frequency (either increasing or decreasing) should consider the free vibration duration and the dynamic characteristics of the annular circular composite plate.

**Figure 5:**
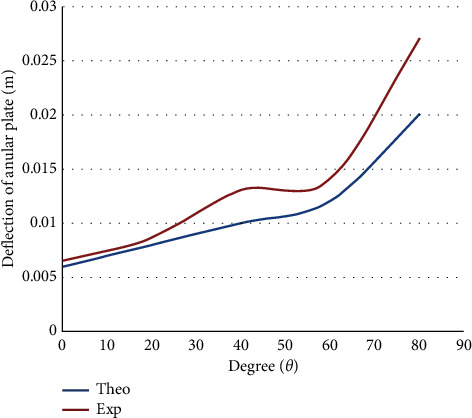
The deflection of an annular circular composite plate against the angle of contact for simply supported.

Figure 5 depicts the movement of the composite plate against the contact angle pertaining to a simply supported scenario. The plate deflection profile rises exponentially with the contact angle pertaining to the orthotropic annular circular plate. The error percentages concerning the conceptual and practical outcomes align well since the orthotropic plate has a very high curve degree.

**Figure 6:**
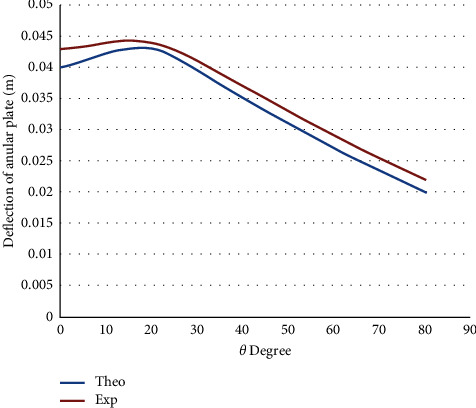
The deflection of the annular circular composite plate against the angle of contact for built-in supported.

Figure 6 shows the deflection of the annular circular composite plate against the angle of contact for built-in support. Increasing of contact angle led to a sinusoidal deflection since contact loading for several points is minimal or nearly fixed. The measured natural frequency was varied based on the boundary conditions. For example, the recorded natural frequency for the simply supported was 750 Hz compared with 875 Hz for the built-in boundary conditions. For built-in boundary criteria, shear stress impact rises because of moments at the ends along with the supporting loads. This transverse stress leads to a higher natural frequency. Typically, theoretical outcomes align well with practical results; consequently, the numerical is impacted by the change in representation concerning impact loading and the peak potential energy that changes with composite material characteristics.

**Figure 7:**
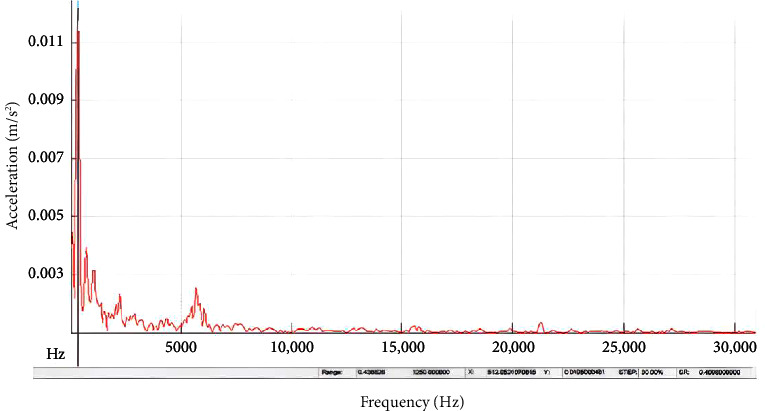
Experimental response spectrum of a simply supported annular circular plate with 40 cm diameter and 20 mm thickness.

**Figure 8:**
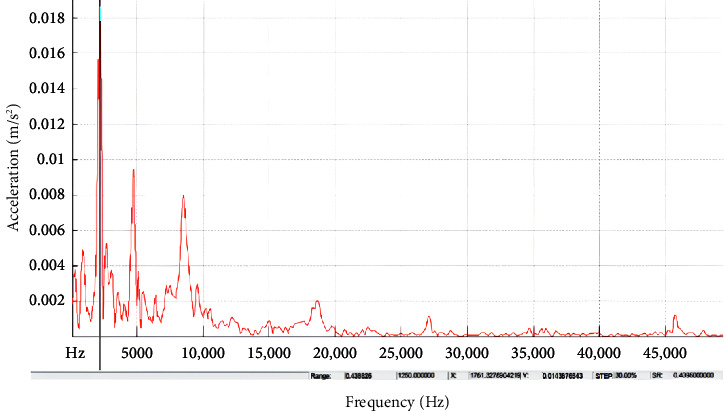
Experimental response spectrum of a built-in supported annular circular plate with 40 cm diameter and 20 mm thickness.

In order to highlight the aspects that have a substantial effect on natural frequency increase pertaining to the annular circular composite plate, Figures 7 and 8 provide precise information concerning the increasing response, specifically for the built-in boundary criteria. In these figures, it can be noticed that the time response for transmitting the impulse waves for high frequency for the built-in annular circular plate when compared with simply supported conditions. It can be attributed these results of the deflections for the annular circular plate to the effect of orientation of the fibers and the volumetric ratio of thecomposite material that led to rise the wave propagation to be more uniform in built-in condition, although the time duration of test for the annular circular composite plate was very short.

## 5. Conclusions

Based on the results obtained, the accuracy and effectiveness of the newly developed mathematical model have been proven, which is capable of calculating the response of a circular plate made of a glass/polyester composite under different boundary conditions. This mathematical model, which has been verified with the results of experimental work, has the ability to overcome the difficulties faced by other researchers, which is considered useful in the process of designing panels that meet the requirements of the industrial sector on the one hand and the customer on the other hand. The main goal is to obtain the best possible performance, taking into account optimal safety within a wide range of vibration frequencies. Finally, it can be summarized the main points that were concluded based on the obtained results as follows:

• The mathematical model has been derived successfully to model the free vibration of the processed annular circular composite plate.
• The mathematical model has been verified efficiently with real experiments by subjecting the fabricated composite plate to a vibration test.
• There was good agreement between theoretical and experimental values particularly for the built-in supported boundary conditions.
• The measured natural frequency for built-in support was greater due to the increasing shear stress impact as a result of moments at the ends along with supporting loads that lead to higher natural frequency compared to simply supported boundary conditions.
• Consequently, the built-in condition generates a higher natural frequency spectrum as the sig view shows.

## Figures and Tables

**Table 1:** Definitions and units of all symbols used in the mathematical modeling.

**Symbol**	**Definition**	**Unit**
*a*_1_	Major distance of ellipse axis	m
*b*_1_	Minor distance of ellipse axis	m
*a*_2_	Difference radius of curvatures between ellipse and semi-circle centers	m
*C*	Particular solution constant	
*D*_*r*_, *D*_*θ*_, and *D*_*rθ*_	Radial, tangential, and twisting flexural rigidity	N·m
*L*	Length of simply supported plate	m
*L*_1_	Difference length between two points of support	m
*m*_1_	Single trigonometric of furrier series	
*m*_*r*_, *m*_*θ*_, and *m*_*rθ*_	Circular plate bending and twisting	N·m
*P*_*o*_	Maximum applied pressure	N/m^2^
*r*, and *θ*	Polar coordinates	m, degree
*r*_1_	Radius of clamped center	m
*q*	Loading contact per unit length	N/m
*w*	Circular plate deflection	m
*X*, *y*	Cartesian coordinates	m
*θ*_1_, *θ*_2_	Angles of the beginning and the ending of clamping	Degree

**Table 2:** Mechanical characteristics of E-glass fiber and polyester resin.

**Material**	**Properties**	**Value**
E-glass fiber	Elasticity modulus (GPa)	74
	Shear modulus (GPa)	30
	Density (kg/m^3^)	2600
	Poisson ratio	0.25

Polyester resin	Elasticity modulus (GPa)	4.0
	Shear modulus (GPa)	1.4
	Density (kg/m^3^)	1200
	Poisson ratio	0.4

## Data Availability

The authors have nothing to report.
